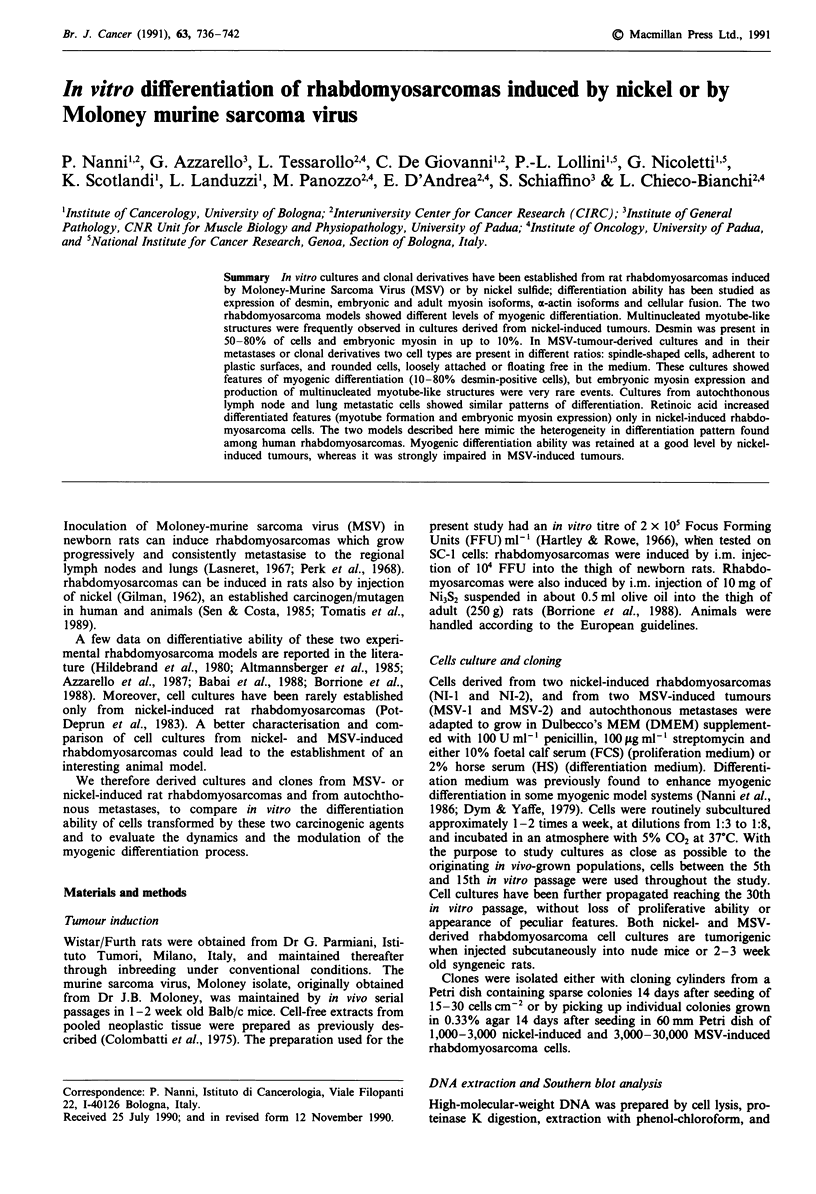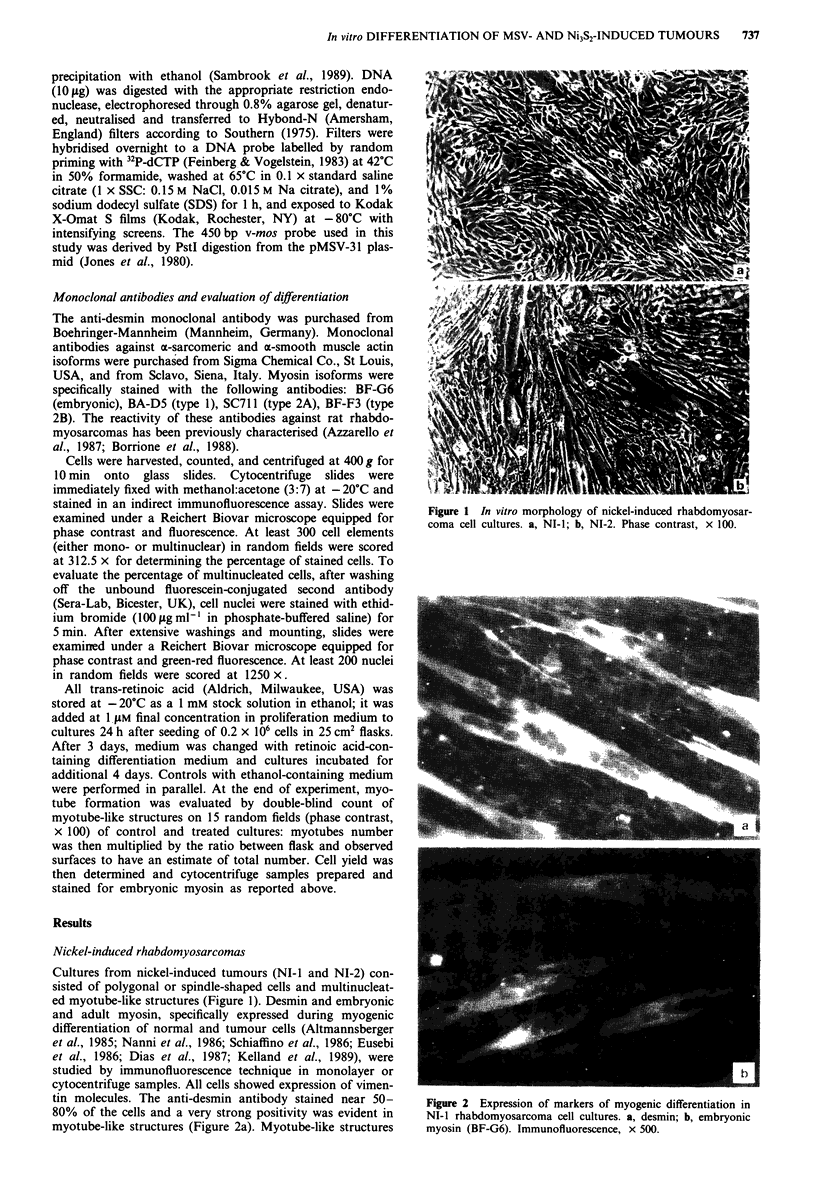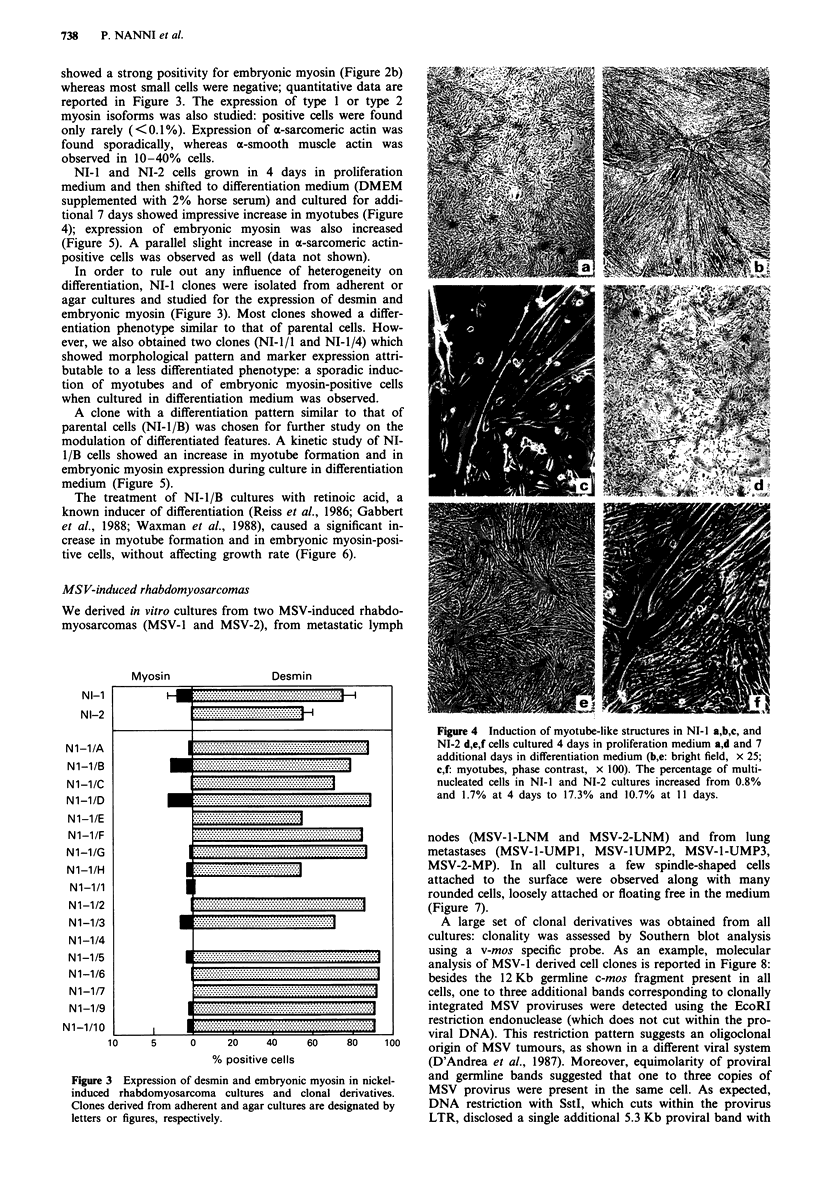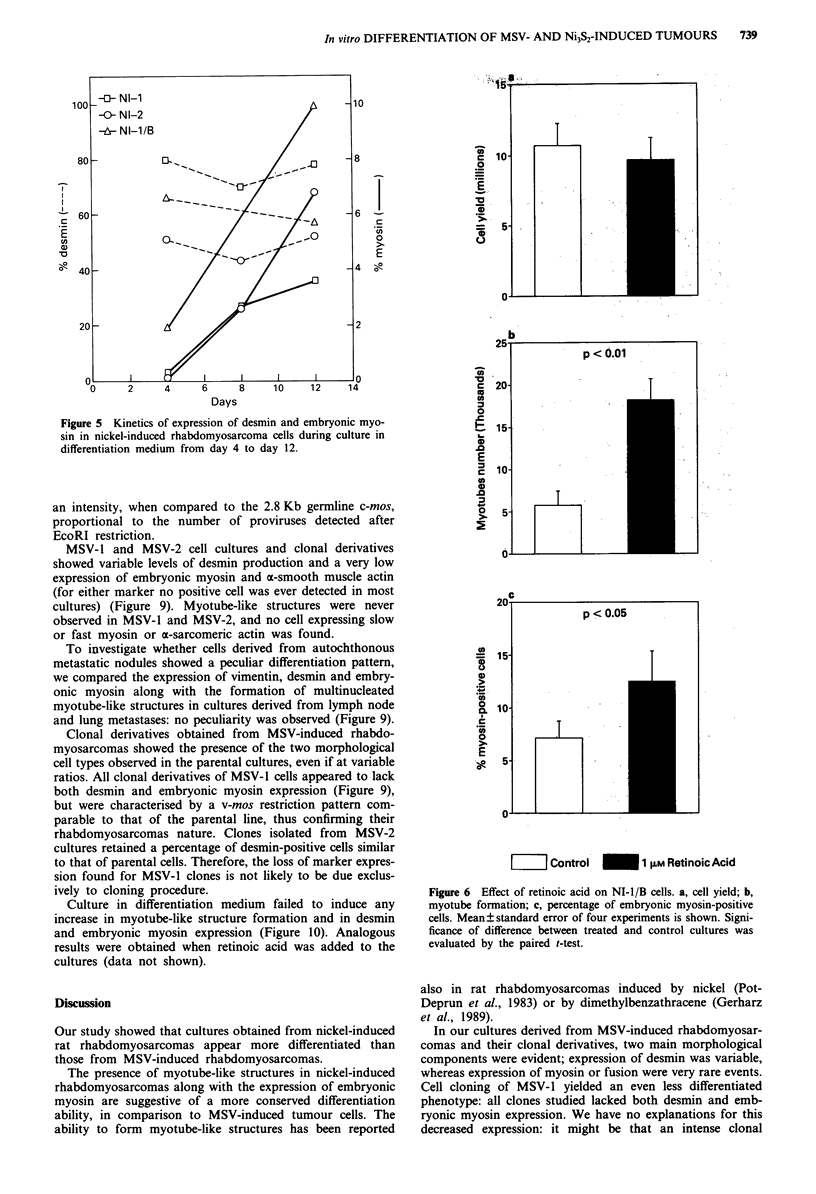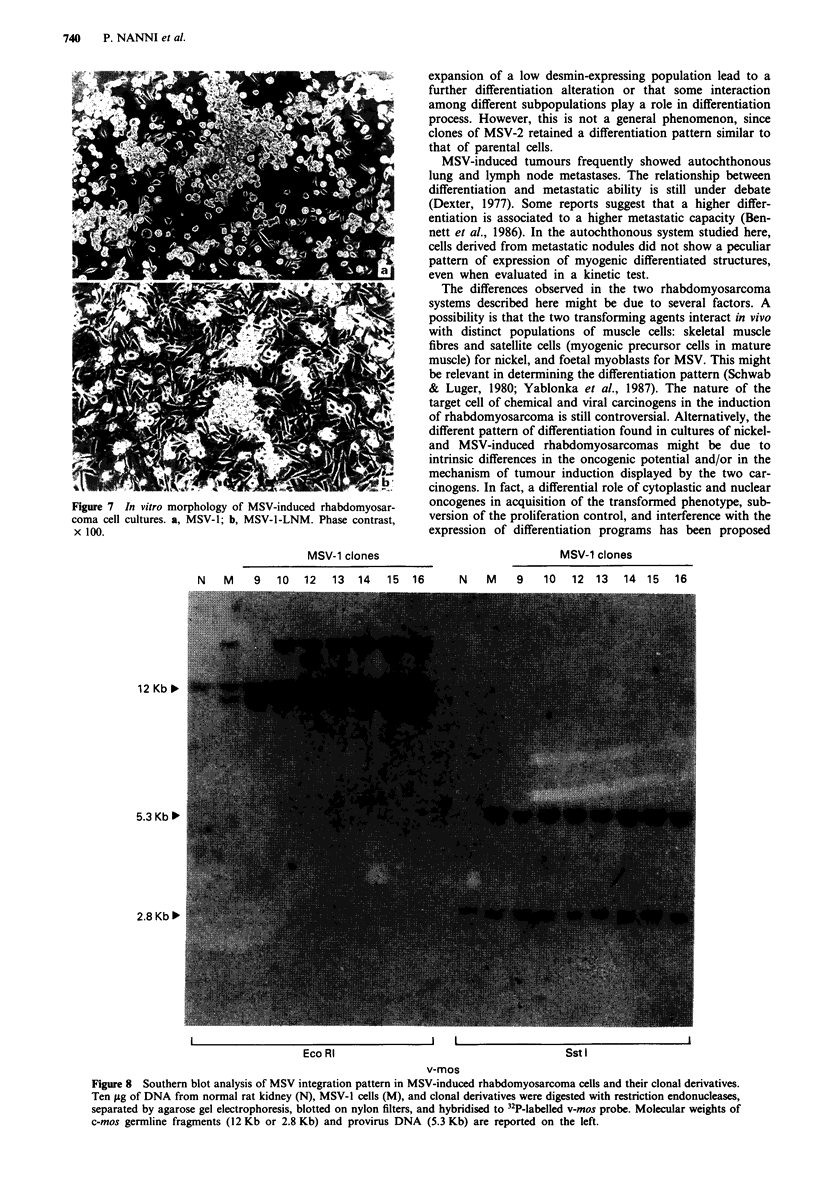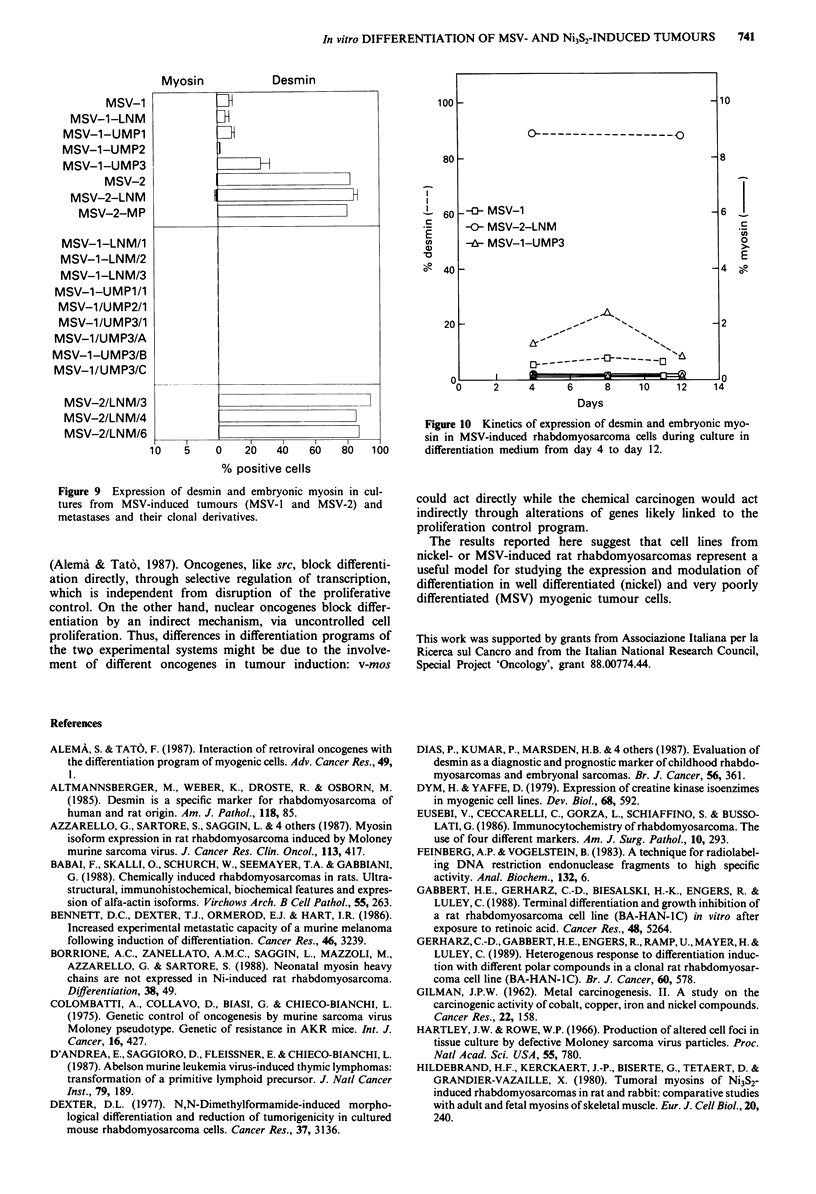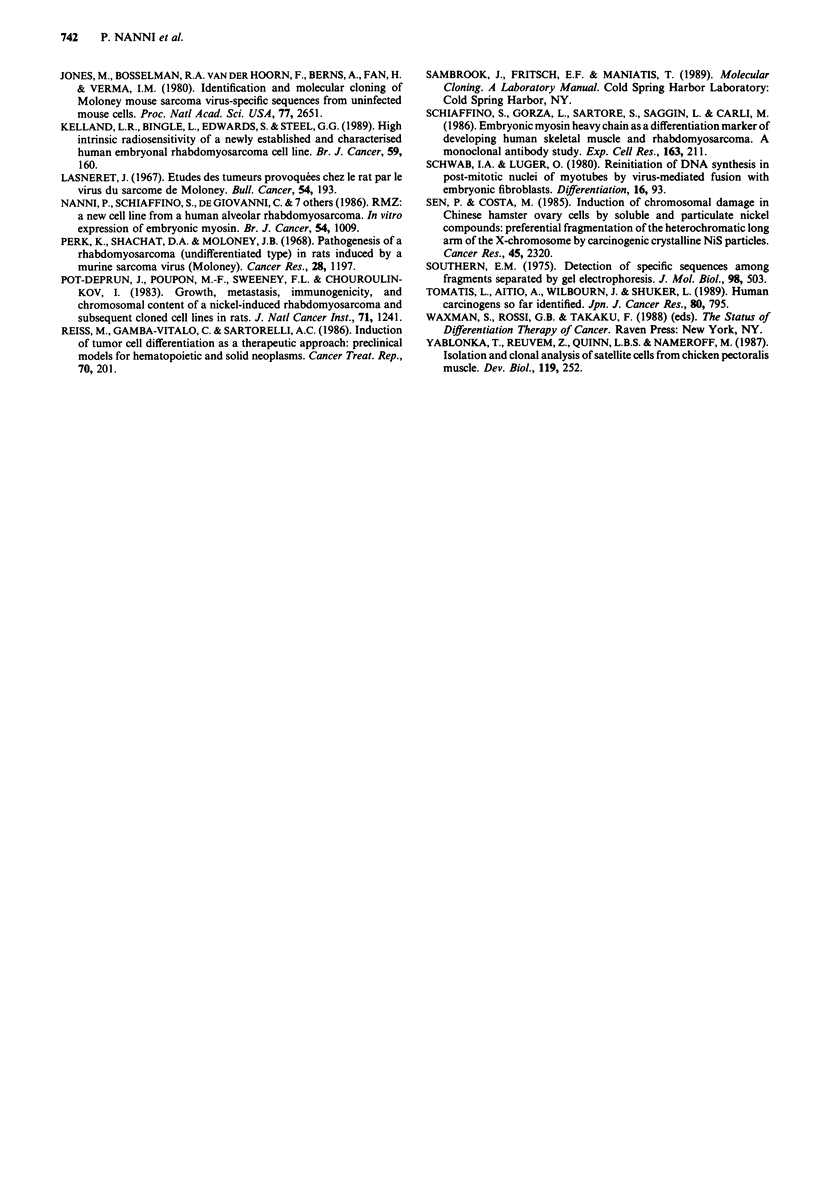# In vitro differentiation of rhabdomyosarcomas induced by nickel or by Moloney murine sarcoma virus.

**DOI:** 10.1038/bjc.1991.165

**Published:** 1991-05

**Authors:** P. Nanni, G. Azzarello, L. Tessarollo, C. De Giovanni, P. L. Lollini, G. Nicoletti, K. Scotlandi, L. Landuzzi, M. Panozzo, E. D'Andrea

**Affiliations:** Institute of Cancerology, University of Bologna, Italy.

## Abstract

**Images:**


Br. J. Cancer (1991), 63, 736–742 © Macmillan Press Ltd., 1991

### *In vitro* differentiation of rhabdomyosarcomas induced by nickel or by Moloney murine sarcoma virus

P. Nanni[1,2], G. Azzarello[3], L. Tessarollo[2,4], C. De Giovanni[1,2], P.-L. Lollini[1,5], G. Nicoletti[1,5], K. Scotlandi[1], L. Landuzzi[1], M. Panozzo[2,4], E. D'Andrea[2,4], S. Schiaffino[3] & L. Chieco-Bianchi[2,4]

[1]*Institute of Cancerology, University of Bologna;* [2]*Interuniversity Center for Cancer Research (CIRC);* [3]*Institute of General Pathology, CNR Unit for Muscle Biology and Physiopathology, University of Padua;* [4]*Institute of Oncology, University of Padua, and* [5]*National Institute for Cancer Research, Genoa, Section of Bologna, Italy.*

**Summary** *In vitro* cultures and clonal derivatives have been established from rat rhabdomyosarcomas induced by Moloney-Murine Sarcoma Virus (MSV) or by nickel sulfide; differentiation ability has been studied as expression of desmin, embryonic and adult myosin isoforms, α-actin isoforms and cellular fusion. The two rhabdomyosarcoma models showed different levels of myogenic differentiation. Multinucleated myotube-like structures were frequently observed in cultures derived from nickel-induced tumours. Desmin was present in 50–80% of cells and embryonic myosin in up to 10%. In MSV-tumour-derived cultures and in their metastases or clonal derivatives two cell types are present in different ratios: spindle-shaped cells, adherent to plastic surfaces, and rounded cells, loosely attached or floating free in the medium. These cultures showed features of myogenic differentiation (10–80% desmin-positive cells), but embryonic myosin expression and production of multinucleated myotube-like structures were very rare events. Cultures from autochthonous lymph node and lung metastatic cells showed similar patterns of differentiation. Retinoic acid increased differentiated features (myotube formation and embryonic myosin expression) only in nickel-induced rhabdomyosarcoma cells. The two models described here mimic the heterogeneity in differentiation pattern found among human rhabdomyosarcomas. Myogenic differentiation ability was retained at a good level by nickel-induced tumours, whereas it was strongly impaired in MSV-induced tumours.

Inoculation of Moloney-murine sarcoma virus (MSV) in newborn rats can induce rhabdomyosarcomas which grow progressively and consistently metastasise to the regional lymph nodes and lungs (Lasneret, 1967; Perk *et al.*, 1968). rhabdomyosarcomas can be induced in rats also by injection of nickel (Gilman, 1962), an established carcinogen/mutagen in human and animals (Sen & Costa, 1985; Tomatis *et al.*, 1989).

A few data on differentiative ability of these two experimental rhabdomyosarcoma models are reported in the literature (Hildebrand *et al.*, 1980; Altmannsberger *et al.*, 1985; Azzarello *et al.*, 1987; Babai *et al.*, 1988; Borrione *et al.*, 1988). Moreover, cell cultures have been rarely established only from nickel-induced rat rhabdomyosarcomas (Pot-Deprun *et al.*, 1983). A better characterisation and comparison of cell cultures from nickel- and MSV-induced rhabdomyosarcomas could lead to the establishment of an interesting animal model.

We therefore derived cultures and clones from MSV- or nickel-induced rat rhabdomyosarcomas and from autochthonous metastases, to compare *in vitro* the differentiation ability of cells transformed by these two carcinogenic agents and to evaluate the dynamics and the modulation of the myogenic differentiation process.

### Materials and methods

### *Tumour induction*

Wistar/Furth rats were obtained from Dr G. Parmiani, Istituto Tumori, Milano, Italy, and maintained thereafter through inbreeding under conventional conditions. The murine sarcoma virus, Moloney isolate, originally obtained from Dr J.B. Moloney, was maintained by *in vivo* serial passages in 1–2 week old Balb/c mice. Cell-free extracts from pooled neoplastic tissue were prepared as previously described (Colombatti *et al.*, 1975). The preparation used for the present study had an *in vitro* titre of $2 \times 10^5$ Focus Forming Units (FFU) $ml^{-1}$ (Hartley & Rowe, 1966), when tested on SC-1 cells: rhabdomyosarcomas were induced by i.m. injection of $10^4$ FFU into the thigh of newborn rats. Rhabdomyosarcomas were also induced by i.m. injection of 10 mg of $Ni_3S_2$ suspended in about 0.5 ml olive oil into the thigh of adult (250 g) rats (Borrione *et al.*, 1988). Animals were handled according to the European guidelines.

### *Cells culture and cloning*

Cells derived from two nickel-induced rhabdomyosarcomas (NI-1 and NI-2), and from two MSV-induced tumours (MSV-1 and MSV-2) and autochthonous metastases were adapted to grow in Dulbecco's MEM (DMEM) supplemented with 100 U $ml^{-1}$ penicillin, 100 $\mu g\ ml^{-1}$ streptomycin and either 10% foetal calf serum (FCS) (proliferation medium) or 2% horse serum (HS) (differentiation medium). Differentiation medium was previously found to enhance myogenic differentiation in some myogenic model systems (Nanni *et al.*, 1986; Dym & Yaffe, 1979). Cells were routinely subcultured approximately 1–2 times a week, at dilutions from 1:3 to 1:8, and incubated in an atmosphere with 5% $CO_2$ at 37°C. With the purpose to study cultures as close as possible to the originating *in vivo*-grown populations, cells between the 5th and 15th *in vitro* passage were used throughout the study. Cell cultures have been further propagated reaching the 30th *in vitro* passage, without loss of proliferative ability or appearance of peculiar features. Both nickel- and MSV-derived rhabdomyosarcoma cell cultures are tumorigenic when injected subcutaneously into nude mice or 2–3 week old syngeneic rats.

Clones were isolated either with cloning cylinders from a Petri dish containing sparse colonies 14 days after seeding of 15–30 cells $cm^{-2}$ or by picking up individual colonies grown in 0.33% agar 14 days after seeding in 60 mm Petri dish of 1,000–3,000 nickel-induced and 3,000–30,000 MSV-induced rhabdomyosarcoma cells.

### *DNA extraction and Southern blot analysis*

High-molecular-weight DNA was prepared by cell lysis, proteinase K digestion, extraction with phenol-chloroform, and

Correspondence: P. Nanni, Istituto di Cancerologia, Viale Filopanti 22, I-40126 Bologna, Italy.
Received 25 July 1990; and in revised form 12 November 1990.

precipitation with ethanol (Sambrook *et al.*, 1989). DNA (10 µg) was digested with the appropriate restriction endonuclease, electrophoresed through 0.8% agarose gel, denatured, neutralised and transferred to Hybond-N (Amersham, England) filters according to Southern (1975). Filters were hybridised overnight to a DNA probe labelled by random priming with $^{32}$P-dCTP (Feinberg & Vogelstein, 1983) at 42°C in 50% formamide, washed at 65°C in 0.1 × standard saline citrate (1 × SSC: 0.15 M NaCl, 0.015 M Na citrate), and 1% sodium dodecyl sulfate (SDS) for 1 h, and exposed to Kodak X-Omat S films (Kodak, Rochester, NY) at −80°C with intensifying screens. The 450 bp v-*mos* probe used in this study was derived by PstI digestion from the pMSV-31 plasmid (Jones *et al.*, 1980).

*Monoclonal antibodies and evaluation of differentiation*

The anti-desmin monoclonal antibody was purchased from Boehringer-Mannheim (Mannheim, Germany). Monoclonal antibodies against α-sarcomeric and α-smooth muscle actin isoforms were purchased from Sigma Chemical Co., St Louis, USA, and from Sclavo, Siena, Italy. Myosin isoforms were specifically stained with the following antibodies: BF-G6 (embryonic), BA-D5 (type 1), SC711 (type 2A), BF-F3 (type 2B). The reactivity of these antibodies against rat rhabdomyosarcomas has been previously characterised (Azzarello *et al.*, 1987; Borrione *et al.*, 1988).

Cells were harvested, counted, and centrifuged at 400 *g* for 10 min onto glass slides. Cytocentrifuge slides were immediately fixed with methanol:acetone (3:7) at −20°C and stained in an indirect immunofluorescence assay. Slides were examined under a Reichert Biovar microscope equipped for phase contrast and fluorescence. At least 300 cell elements (either mono- or multinuclear) in random fields were scored at 312.5 × for determining the percentage of stained cells. To evaluate the percentage of multinucleated cells, after washing off the unbound fluorescein-conjugated second antibody (Sera-Lab, Bicester, UK), cell nuclei were stained with ethidium bromide (100 µg ml$^{-1}$ in phosphate-buffered saline) for 5 min. After extensive washings and mounting, slides were examined under a Reichert Biovar microscope equipped for phase contrast and green-red fluorescence. At least 200 nuclei in random fields were scored at 1250 ×.

All trans-retinoic acid (Aldrich, Milwaukee, USA) was stored at −20°C as a 1 mM stock solution in ethanol; it was added at 1 µM final concentration in proliferation medium to cultures 24 h after seeding of 0.2 × 10$^6$ cells in 25 cm$^2$ flasks. After 3 days, medium was changed with retinoic acid-containing differentiation medium and cultures incubated for additional 4 days. Controls with ethanol-containing medium were performed in parallel. At the end of experiment, myotube formation was evaluated by double-blind count of myotube-like structures on 15 random fields (phase contrast, × 100) of control and treated cultures: myotubes number was then multiplied by the ratio between flask and observed surfaces to have an estimate of total number. Cell yield was then determined and cytocentrifuge samples prepared and stained for embryonic myosin as reported above.

### Results

*Nickel-induced rhabdomyosarcomas*

Cultures from nickel-induced tumours (NI-1 and NI-2) consisted of polygonal or spindle-shaped cells and multinucleated myotube-like structures (Figure 1). Desmin and embryonic and adult myosin, specifically expressed during myogenic differentiation of normal and tumour cells (Altmannsberger *et al.*, 1985; Nanni *et al.*, 1986; Schiaffino *et al.*, 1986; Eusebi *et al.*, 1986; Dias *et al.*, 1987; Kelland *et al.*, 1989), were studied by immunofluorescence technique in monolayer or cytocentrifuge samples. All cells showed expression of vimentin molecules. The anti-desmin antibody stained near 50–80% of the cells and a very strong positivity was evident in myotube-like structures (Figure 2a). Myotube-like structures

**Figure 1** *In vitro* morphology of nickel-induced rhabdomyosarcoma cell cultures. **a**, NI-1; **b**, NI-2. Phase contrast, × 100.

**Figure 2** Expression of markers of myogenic differentiation in NI-1 rhabdomyosarcoma cell cultures. **a**, desmin; **b**, embryonic myosin (BF-G6). Immunofluorescence, × 500.

showed a strong positivity for embryonic myosin (Figure 2b) whereas most small cells were negative; quantitative data are reported in Figure 3. The expression of type 1 or type 2 myosin isoforms was also studied: positive cells were found only rarely (<0.1%). Expression of α-sarcomeric actin was found sporadically, whereas α-smooth muscle actin was observed in 10–40% cells.

NI-1 and NI-2 cells grown in 4 days in proliferation medium and then shifted to differentiation medium (DMEM supplemented with 2% horse serum) and cultured for additional 7 days showed impressive increase in myotubes (Figure 4); expression of embryonic myosin was also increased (Figure 5). A parallel slight increase in α-sarcomeric actin-positive cells was observed as well (data not shown).

In order to rule out any influence of heterogeneity on differentiation, NI-1 clones were isolated from adherent or agar cultures and studied for the expression of desmin and embryonic myosin (Figure 3). Most clones showed a differentiation phenotype similar to that of parental cells. However, we also obtained two clones (NI-1/1 and NI-1/4) which showed morphological pattern and marker expression attributable to a less differentiated phenotype: a sporadic induction of myotubes and of embryonic myosin-positive cells when cultured in differentiation medium was observed.

A clone with a differentiation pattern similar to that of parental cells (NI-1/B) was chosen for further study on the modulation of differentiated features. A kinetic study of NI-1/B cells showed an increase in myotube formation and in embryonic myosin expression during culture in differentiation medium (Figure 5).

The treatment of NI-1/B cultures with retinoic acid, a known inducer of differentiation (Reiss *et al.*, 1986; Gabbert *et al.*, 1988; Waxman *et al.*, 1988), caused a significant increase in myotube formation and in embryonic myosin-positive cells, without affecting growth rate (Figure 6).

### *MSV-induced rhabdomyosarcomas*

We derived *in vitro* cultures from two MSV-induced rhabdomyosarcomas (MSV-1 and MSV-2), from metastatic lymph nodes (MSV-1-LNM and MSV-2-LNM) and from lung metastases (MSV-1-UMP1, MSV-1UMP2, MSV-1-UMP3, MSV-2-MP). In all cultures a few spindle-shaped cells attached to the surface were observed along with many rounded cells, loosely attached or floating free in the medium (Figure 7).

A large set of clonal derivatives was obtained from all cultures: clonality was assessed by Southern blot analysis using a v-*mos* specific probe. As an example, molecular analysis of MSV-1 derived cell clones is reported in Figure 8: besides the 12 Kb germline c-*mos* fragment present in all cells, one to three additional bands corresponding to clonally integrated MSV proviruses were detected using the EcoRI restriction endonuclease (which does not cut within the proviral DNA). This restriction pattern suggests an oligoclonal origin of MSV tumours, as shown in a different viral system (D'Andrea *et al.*, 1987). Moreover, equimolarity of proviral and germline bands suggested that one to three copies of MSV provirus were present in the same cell. As expected, DNA restriction with SstI, which cuts within the provirus LTR, disclosed a single additional 5.3 Kb proviral band with

**Figure 3** Expression of desmin and embryonic myosin in nickel-induced rhabdomyosarcoma cultures and clonal derivatives. Clones derived from adherent and agar cultures are designated by letters or figures, respectively.

**Figure 4** Induction of myotube-like structures in NI-1 **a,b,c**, and NI-2 **d,e,f** cells cultured 4 days in proliferation medium **a,d** and 7 additional days in differentiation medium (**b,e**: bright field, × 25; **c,f**: myotubes, phase contrast, × 100). The percentage of multinucleated cells in NI-1 and NI-2 cultures increased from 0.8% and 1.7% at 4 days to 17.3% and 10.7% at 11 days.

**Figure 5** Kinetics of expression of desmin and embryonic myosin in nickel-induced rhabdomyosarcoma cells during culture in differentiation medium from day 4 to day 12.

an intensity, when compared to the 2.8 Kb germline c-*mos*, proportional to the number of proviruses detected after EcoRI restriction.

MSV-1 and MSV-2 cell cultures and clonal derivatives showed variable levels of desmin production and a very low expression of embryonic myosin and α-smooth muscle actin (for either marker no positive cell was ever detected in most cultures) (Figure 9). Myotube-like structures were never observed in MSV-1 and MSV-2, and no cell expressing slow or fast myosin or α-sarcomeric actin was found.

To investigate whether cells derived from autochthonous metastatic nodules showed a peculiar differentiation pattern, we compared the expression of vimentin, desmin and embryonic myosin along with the formation of multinucleated myotube-like structures in cultures derived from lymph node and lung metastases: no peculiarity was observed (Figure 9).

Clonal derivatives obtained from MSV-induced rhabdomyosarcomas showed the presence of the two morphological cell types observed in the parental cultures, even if at variable ratios. All clonal derivatives of MSV-1 cells appeared to lack both desmin and embryonic myosin expression (Figure 9), but were characterised by a v-*mos* restriction pattern comparable to that of the parental line, thus confirming their rhabdomyosarcomas nature. Clones isolated from MSV-2 cultures retained a percentage of desmin-positive cells similar to that of parental cells. Therefore, the loss of marker expression found for MSV-1 clones is not likely to be due exclusively to cloning procedure.

Culture in differentiation medium failed to induce any increase in myotube-like structure formation and in desmin and embryonic myosin expression (Figure 10). Analogous results were obtained when retinoic acid was added to the cultures (data not shown).

### Discussion

Our study showed that cultures obtained from nickel-induced rat rhabdomyosarcomas appear more differentiated than those from MSV-induced rhabdomyosarcomas.

The presence of myotube-like structures in nickel-induced rhabdomyosarcomas along with the expression of embryonic myosin are suggestive of a more conserved differentiation ability, in comparison to MSV-induced tumour cells. The ability to form myotube-like structures has been reported

**Figure 6** Effect of retinoic acid on NI-1/B cells. **a**, cell yield; **b**, myotube formation; **c**, percentage of embryonic myosin-positive cells. Mean ± standard error of four experiments is shown. Significance of difference between treated and control cultures was evaluated by the paired *t*-test.

also in rat rhabdomyosarcomas induced by nickel (Pot-Deprun *et al.*, 1983) or by dimethylbenzathracene (Gerharz *et al.*, 1989).

In our cultures derived from MSV-induced rhabdomyosarcomas and their clonal derivatives, two main morphological components were evident; expression of desmin was variable, whereas expression of myosin or fusion were very rare events. Cell cloning of MSV-1 yielded an even less differentiated phenotype: all clones studied lacked both desmin and embryonic myosin expression. We have no explanations for this decreased expression: it might be that an intense clonal

Figure 7 *In vitro* morphology of MSV-induced rhabdomyosarcoma cell cultures. a, MSV-1; b, MSV-1-LNM. Phase contrast, × 100.

expansion of a low desmin-expressing population lead to a further differentiation alteration or that some interaction among different subpopulations play a role in differentiation process. However, this is not a general phenomenon, since clones of MSV-2 retained a differentiation pattern similar to that of parental cells.

MSV-induced tumours frequently showed autochthonous lung and lymph node metastases. The relationship between differentiation and metastatic ability is still under debate (Dexter, 1977). Some reports suggest that a higher differentiation is associated to a higher metastatic capacity (Bennett *et al.*, 1986). In the autochthonous system studied here, cells derived from metastatic nodules did not show a peculiar pattern of expression of myogenic differentiated structures, even when evaluated in a kinetic test.

The differences observed in the two rhabdomyosarcoma systems described here might be due to several factors. A possibility is that the two transforming agents interact *in vivo* with distinct populations of muscle cells: skeletal muscle fibres and satellite cells (myogenic precursor cells in mature muscle) for nickel, and foetal myoblasts for MSV. This might be relevant in determining the differentiation pattern (Schwab & Luger, 1980; Yablonka *et al.*, 1987). The nature of the target cell of chemical and viral carcinogens in the induction of rhabdomyosarcoma is still controversial. Alternatively, the different pattern of differentiation found in cultures of nickel- and MSV-induced rhabdomyosarcomas might be due to intrinsic differences in the oncogenic potential and/or in the mechanism of tumour induction displayed by the two carcinogens. In fact, a differential role of cytoplastic and nuclear oncogenes in acquisition of the transformed phenotype, subversion of the proliferation control, and interference with the expression of differentiation programs has been proposed

Figure 8 Southern blot analysis of MSV integration pattern in MSV-induced rhabdomyosarcoma cells and their clonal derivatives. Ten μg of DNA from normal rat kidney (N), MSV-1 cells (M), and clonal derivatives were digested with restriction endonucleases, separated by agarose gel electrophoresis, blotted on nylon filters, and hybridised to $^{32}$P-labelled v-*mos* probe. Molecular weights of c-*mos* germline fragments (12 Kb or 2.8 Kb) and provirus DNA (5.3 Kb) are reported on the left.

**Figure 9** Expression of desmin and embryonic myosin in cultures from MSV-induced tumours (MSV-1 and MSV-2) and metastases and their clonal derivatives.

**Figure 10** Kinetics of expression of desmin and embryonic myosin in MSV-induced rhabdomyosarcoma cells during culture in differentiation medium from day 4 to day 12.

(Alemà & Tatò, 1987). Oncogenes, like *src*, block differentiation directly, through selective regulation of transcription, which is independent from disruption of the proliferative control. On the other hand, nuclear oncogenes block differentiation by an indirect mechanism, via uncontrolled cell proliferation. Thus, differences in differentiation programs of the two experimental systems might be due to the involvement of different oncogenes in tumour induction: v-*mos* could act directly while the chemical carcinogen would act indirectly through alterations of genes likely linked to the proliferation control program.

The results reported here suggest that cell lines from nickel- or MSV-induced rat rhabdomyosarcomas represent a useful model for studying the expression and modulation of differentiation in well differentiated (nickel) and very poorly differentiated (MSV) myogenic tumour cells.

This work was supported by grants from Associazione Italiana per la Ricerca sul Cancro and from the Italian National Research Council, Special Project 'Oncology', grant 88.00774.44.

### References

ALEMÀ, S. & TATÒ, F. (1987). Interaction of retroviral oncogenes with the differentiation program of myogenic cells. *Adv. Cancer Res.*, **49**, 1.

ALTMANNSBERGER, M., WEBER, K., DROSTE, R. & OSBORN, M. (1985). Desmin is a specific marker for rhabdomyosarcoma of human and rat origin. *Am. J. Pathol.*, **118**, 85.

AZZARELLO, G., SARTORE, S., SAGGIN, L. & 4 others (1987). Myosin isoform expression in rat rhabdomyosarcoma induced by Moloney murine sarcoma virus. *J. Cancer Res. Clin. Oncol.*, **113**, 417.

BABAI, F., SKALLI, O., SCHURCH, W., SEEMAYER, T.A. & GABBIANI, G. (1988). Chemically induced rhabdomyosarcomas in rats. Ultrastructural, immunohistochemical, biochemical features and expression of alfa-actin isoforms. *Virchows Arch. B Cell Pathol.*, **55**, 263.

BENNETT, D.C., DEXTER, T.J., ORMEROD, E.J. & HART, I.R. (1986). Increased experimental metastatic capacity of a murine melanoma following induction of differentiation. *Cancer Res.*, **46**, 3239.

BORRIONE, A.C., ZANELLATO, A.M.C., SAGGIN, L., MAZZOLI, M., AZZARELLO, G. & SARTORE, S. (1988). Neonatal myosin heavy chains are not expressed in Ni-induced rat rhabdomyosarcoma. *Differentiation*, **38**, 49.

COLOMBATTI, A., COLLAVO, D., BIASI, G. & CHIECO-BIANCHI, L. (1975). Genetic control of oncogenesis by murine sarcoma virus Moloney pseudotype. Genetic of resistance in AKR mice. *Int. J. Cancer*, **16**, 427.

D'ANDREA, E., SAGGIORO, D., FLEISSNER, E. & CHIECO-BIANCHI, L. (1987). Abelson murine leukemia virus-induced thymic lymphomas: transformation of a primitive lymphoid precursor. *J. Natl Cancer Inst.*, **79**, 189.

DEXTER, D.L. (1977). N,N-Dimethylformamide-induced morphological differentiation and reduction of tumorigenicity in cultured mouse rhabdomyosarcoma cells. *Cancer Res.*, **37**, 3136.

DIAS, P., KUMAR, P., MARSDEN, H.B. & 4 others (1987). Evaluation of desmin as a diagnostic and prognostic marker of childhood rhabdomyosarcomas and embryonal sarcomas. *Br. J. Cancer*, **56**, 361.

DYM, H. & YAFFE, D. (1979). Expression of creatine kinase isoenzimes in myogenic cell lines. *Dev. Biol.*, **68**, 592.

EUSEBI, V., CECCARELLI, C., GORZA, L., SCHIAFFINO, S. & BUSSOLATI, G. (1986). Immunocytochemistry of rhabdomyosarcoma. The use of four different markers. *Am. J. Surg. Pathol.*, **10**, 293.

FEINBERG, A.P. & VOGELSTEIN, B. (1983). A technique for radiolabeling DNA restriction endonuclease fragments to high specific activity. *Anal. Biochem.*, **132**, 6.

GABBERT, H.E., GERHARZ, C.-D., BIESALSKI, H.-K., ENGERS, R. & LULEY, C. (1988). Terminal differentiation and growth inhibition of a rat rhabdomyosarcoma cell line (BA-HAN-1C) *in vitro* after exposure to retinoic acid. *Cancer Res.*, **48**, 5264.

GERHARZ, C.-D., GABBERT, H.E., ENGERS, R., RAMP, U., MAYER, H. & LULEY, C. (1989). Heterogenous response to differentiation induction with different polar compounds in a clonal rat rhabdomyosarcoma cell line (BA-HAN-1C). *Br. J. Cancer*, **60**, 578.

GILMAN, J.P.W. (1962). Metal carcinogenesis. II. A study on the carcinogenic activity of cobalt, copper, iron and nickel compounds. *Cancer Res.*, **22**, 158.

HARTLEY, J.W. & ROWE, W.P. (1966). Production of altered cell foci in tissue culture by defective Moloney sarcoma virus particles. *Proc. Natl Acad. Sci. USA*, **55**, 780.

HILDEBRAND, H.F., KERCKAERT, J.-P., BISERTE, G., TETAERT, D. & GRANDIER-VAZAILLE, X. (1980). Tumoral myosins of $Ni_3S_2$-induced rhabdomyosarcomas in rat and rabbit: comparative studies with adult and fetal myosins of skeletal muscle. *Eur. J. Cell Biol.*, **20**, 240.

JONES, M., BOSSELMAN, R.A. VAN DER HOORN, F., BERNS, A., FAN, H. & VERMA, I.M. (1980). Identification and molecular cloning of Moloney mouse sarcoma virus-specific sequences from uninfected mouse cells. *Proc. Natl Acad. Sci. USA*, **77**, 2651.

KELLAND, L.R., BINGLE, L., EDWARDS, S. & STEEL, G.G. (1989). High intrinsic radiosensitivity of a newly established and characterised human embryonal rhabdomyosarcoma cell line. *Br. J. Cancer*, **59**, 160.

LASNERET, J. (1967). Etudes des tumeurs provoquées chez le rat par le virus du sarcome de Moloney. *Bull. Cancer*, **54**, 193.

NANNI, P., SCHIAFFINO, S., DE GIOVANNI, C. & 7 others (1986). RMZ: a new cell line from a human alveolar rhabdomyosarcoma. *In vitro* expression of embryonic myosin. *Br. J. Cancer*, **54**, 1009.

PERK, K., SHACHAT, D.A. & MOLONEY, J.B. (1968). Pathogenesis of a rhabdomyosarcoma (undifferentiated type) in rats induced by a murine sarcoma virus (Moloney). *Cancer Res.*, **28**, 1197.

POT-DEPRUN, J., POUPON, M.-F., SWEENEY, F.L. & CHOUROULINKOV, I. (1983). Growth, metastasis, immunogenicity, and chromosomal content of a nickel-induced rhabdomyosarcoma and subsequent cloned cell lines in rats. *J. Natl Cancer Inst.*, **71**, 1241.

REISS, M., GAMBA-VITALO, C. & SARTORELLI, A.C. (1986). Induction of tumor cell differentiation as a therapeutic approach: preclinical models for hematopoietic and solid neoplasms. *Cancer Treat. Rep.*, **70**, 201.

SAMBROOK, J., FRITSCH, E.F. & MANIATIS, T. (1989). *Molecular Cloning. A Laboratory Manual.* Cold Spring Harbor Laboratory: Cold Spring Harbor, NY.

SCHIAFFINO, S., GORZA, L., SARTORE, S., SAGGIN, L. & CARLI, M. (1986). Embryonic myosin heavy chain as a differentiation marker of developing human skeletal muscle and rhabdomyosarcoma. A monoclonal antibody study. *Exp. Cell Res.*, **163**, 211.

SCHWAB, I.A. & LUGER, O. (1980). Reinitiation of DNA synthesis in post-mitotic nuclei of myotubes by virus-mediated fusion with embryonic fibroblasts. *Differentiation*, **16**, 93.

SEN, P. & COSTA, M. (1985). Induction of chromosomal damage in Chinese hamster ovary cells by soluble and particulate nickel compounds: preferential fragmentation of the heterochromatic long arm of the X-chromosome by carcinogenic crystalline NiS particles. *Cancer Res.*, **45**, 2320.

SOUTHERN, E.M. (1975). Detection of specific sequences among fragments separated by gel electrophoresis. *J. Mol. Biol.*, **98**, 503.

TOMATIS, L., AITIO, A., WILBOURN, J. & SHUKER, L. (1989). Human carcinogens so far identified. *Jpn. J. Cancer Res.*, **80**, 795.

WAXMAN, S., ROSSI, G.B. & TAKAKU, F. (1988) (eds). *The Status of Differentiation Therapy of Cancer*. Raven Press: New York, NY.

YABLONKA, T., REUVEM, Z., QUINN, L.B.S. & NAMEROFF, M. (1987). Isolation and clonal analysis of satellite cells from chicken pectoralis muscle. *Dev. Biol.*, **119**, 252.